# Editorial: Unusual Clinical Presentation of COVID-19

**DOI:** 10.3390/jcm11112953

**Published:** 2022-05-24

**Authors:** Pierpaolo Di Micco

**Affiliations:** UOC Medicina, Ospedale Buon Consiglio Fatebenefratelli di Napoli, 80122 Naples, Italy; pdimicco@libero.it

Nearly two years ago, the SARS-CoV2 outbreak began, and our lives have changed significantly since then. The increasing number of articles and other submissions to the scientific literature from around the world has contributed to our knowledge of this virus and its related infectious diseases. The pandemic is still ongoing, although with different clinical approaches because of a series of new insights. Aside from the personal cautions and environmental methods of ameliorating the pandemic (e.g., face masks, reducing access to and time spent in public areas, frequent cleaning of the hands, and the climatic variation and circulation of the virus), the progression of vaccination campaigns reduced the number of infected patients with severe lung failure, as well as the morbidity, mortality, and hospitalization rates for COVID-19. However, there are still several problems with the management of subjects affected by COVID-19 particularly when hospitalization is required, and this clinical trend is even changing the submission time of this Special Issue that reflects the different forms of the clinical presentation of COVID-19.

The first waves of COVID-19 were characterized by the association of lung failure due to interstitial pneumonia with pulmonary embolisms or bacterial/fungal over-infection; detected increased inflammatory markers acted also as prognostic markers and also as targets for pharmacological treatment (e.g., IL-6). In these first phases of the pandemic, the useful role of low molecular weight heparin and enoxaparin has been underlined in several reports; following analyses were performed also in patients with an increased thrombotic risk of developing VTE per se as oncological patients and this item was reported by by Imbalzano et al. [1]; furthermore also the role of dyslipidemias as comorbidities inducing increased cardiovascular events in inpatients with COVID-19 has been reported [2,3]. As for other virus, SARS CoV2 quickly began its cycle of genome mutations that might have induced its prolonged survival against the immunization of the general population. For this reason, several viral variants have been identified in these last 2 years: B.1.1.7 (Alpha), B.1.351 (Beta), P.1 (Gamma), B.1.617.2 (Delta), B.1.427, B.1.429 (Epsilon), P.2 (Zeta), B.1.525 (Eta), P.3 (Theta), B.1.526 (Iota), B.1.617.1 (Kappa), and the recently identified B.1.1.529 (Omicron), all of which have been suggested as the culprits responsible for the prolongation of the SARS CoV2 outbreak and named Variants of concern (VOCs). From a clinical point of view, VOCs may be responsible for the early misidentification of the SARS CoV2 infection using nasopharyngeal swab (NPS). Therefore, if the clinical suspect of COVID-19 is also NPS negative, then the SARS research on other biological fluids or tissues such as bronchoalveolar lavage or urine has been suggested [4]. Furthermore, particularly for hospital workers, the chances of having COVID-19 or a COVID-like syndrome in the presence of suspected signs and symptoms when repeated NPS did not reveal the presence of SARS CoV2 has been underlined by Di Micco et al. [5].

Moreover, regarding the unusual clinical presentation during infection by SARS CoV2, several neuropathological findings have been described including anosmia and/or ageusia, but different neurological signs and symptoms have also been reported, such as depression, insomnia, and tremors/movement disorders [6,7].

Furthermore, damage to the gastrointestinal system, particularly to the pancreas, has been reported in several cohorts of patients [8,9]. These findings may be related to the fact that the expression of the ACE protein may vary in organs such as the pancreas, so the appearance of pancreatitis may be due to this pathophysiological aspect.

Yet, the clinical features of COVID-19 are different than pneumonia and should not be confused with post COVID. Post COVID syndrome, in fact, is a complex clinical condition in which damages due to the acute infection by SARS CoV2 are present for longer than expected, or where other types of damage are present after the recovery from acute infection and are related to chronic dysfunction (e.g., lung dysfunction) [10] or side effects of several drugs used during the acute phase of the disease.

Therefore, after more than 2 years, infection by SARS CoV2 is still ongoing and thus offers us the chance to learn a great deal in different areas, such as respiratory infections, other sites of viral damages, life threatening complications such as bacterial or fungal over-infection and/or venous thromboembolism, and post infection dysfunctions, and we are eager to have the opportunity to improve our clinical and scientific knowledge about these areas. The aim of this editorial for the related Special Issue on the Unusual Clinical Presentation of COVID-19 has also changed, because the collection must work in parallel with pandemic management.
